# CNS distribution, signalling properties and central effects of G-protein coupled receptor 4

**DOI:** 10.1016/j.neuropharm.2018.06.007

**Published:** 2018-08

**Authors:** P.S. Hosford, V. Mosienko, K. Kishi, G. Jurisic, K. Seuwen, B. Kinzel, M.G. Ludwig, J.A. Wells, I.N. Christie, L. Koolen, A.P. Abdala, B.H. Liu, A.V. Gourine, A.G. Teschemacher, S. Kasparov

**Affiliations:** a Physiology, Pharmacology and Neuroscience, University of Bristol, BS8 1TD, UK; b Centre for Cardiovascular and Metabolic Neuroscience, Neuroscience, Physiology and Pharmacology, University College London, WC1E 6BT, UK; c Novartis Institutes for Biomedical Research, CH-4002 Basel, Switzerland; d Baltic Federal University, Kaliningrad 236041, Russian Federation

**Keywords:** GPR4, Antagonist, Lactate, Modulation, Respiration, Distribution

## Abstract

Information on the distribution and biology of the G-protein coupled receptor 4 (GPR4) in the brain is limited. It is currently thought that GPR4 couples to G_s_ proteins and may mediate central respiratory sensitivity to CO_2_. Using a knock-in mouse model, abundant GPR4 expression was detected in the cerebrovascular endothelium and neurones of dorsal raphe, retro-trapezoidal nucleus locus coeruleus and lateral septum. A similar distribution was confirmed using RNAscope in situ hybridisation. In HEK293 cells, overexpressing GPR4, it was highly constitutively active at neutral pH with little further increase in cAMP towards acidic pH. The GPR4 antagonist NE 52-QQ57 effectively blocked GPR4-mediated cAMP accumulation (IC_50_ 26.8 nM in HEK293 cells). In HUVEC which natively express GPR4, physiological acidification (pH 7.4–7.0) resulted in a cAMP increase by ∼55% which was completely prevented by 1 μM NE 52-QQ57. The main extracellular organic acid, l-lactic acid (LL; 1–10 mM), suppressed pH dependent activation of GPR4 in HEK293 and HUVEC cells, suggesting allosteric negative modulation. In unanaesthetised mice and rats, NE 52-QQ57 (20 mg kg^−1^) reduced ventilatory response to 5 and 10% CO_2_. In anaesthetised rats, systemic administration of NE 52-QQ57 (up to 20 mg kg^−1^) had no effect on hemodynamics, cerebral blood flow and blood oxygen level dependent responses. Central administration of NE 52-QQ57 (1 mM) in vagotomised anaesthetised rats did not affect CO_2_-induced respiratory responses. Our results indicate that GPR4 is expressed by multiple neuronal populations and endothelium and that its pH sensitivity is affected by level of expression and LL. NE 52-QQ57 blunts hypercapnic response to CO_2_ but this effect is absent under anaesthesia, possibly due to the inhibitory effect of LL on GPR4.

## 1 Introduction

G-protein coupled receptors (GPCR) represent the largest family of drug targets and, for this reason, are widely medically exploited. Since the publication of the human genome, numerous new putative GPCRs were identified, many without any known endogenous ligands. Such “orphan” GPCR are often still known under the numbers they were given initially. GPR4 was de-orphanized in 2003 when it was found to be highly sensitive to changes in extracellular proton concentration (Ludwig et al., 2003). Since that time, further proton-sensing GPCRs were identified, namely GPR65 and GPR68 (http://www.guidetopharmacology.org).

The cellular phenotype best established to be associated with strong GPR4 expression is endothelium in peripheral tissues as well as in the brain (Huang et al., 2007; Kim et al., 2005; Qiao et al., 2006; Sun et al., 2016; Wyder et al., 2011), this being consistent with the published transcriptome data (Zhang et al., 2014, 2016). GPR4 activity in peripheral endothelial cells has been implicated in blood vessel formation (Dong et al., 2013), transmigration of monocytes (Huang et al., 2007), responses to vascular endothelial growth factor (Wyder et al., 2011), vascular inflammation (Dong et al., 2013) and endothelial adhesiveness (Chen et al., 2011). Strong GPR4 expression was also shown in the kidney where it contributes to the control of acid-base balance (Brown and Wagner, 2012). However, a recent study (Kumar et al., 2015)/id using in situ hybridization did not detect GPR4 in the cerebrovascular endothelium but, instead, almost exclusively in neurones of the brainstem retrotrapezoid nucleus (RTN), an area known to be important for central respiratory CO_2_ chemosensitivity and control of breathing. At the same time, single cell PCR demonstrated strong GPR4 expression in the RTN neurones, in the adjacent C1 catecholaminergic neurones (which generate sympathetic tone (Marina et al., 2013);) and in serotonergic raphe nucleus neurones (Kumar et al., 2015). That study also found in mice GPR4 deletion was associated with reduced respiratory CO_2_ sensitivity implicating GPR4 expressed at RTN neurones in central chemosensitivity to CO_2_ (Kumar et al., 2015).

Thus, there is a certain controversy regarding localization of GPR4 in the brain, especially given recent direct demonstration of GPR4 expression in the capillaries of at least some parts of the brain (Sun et al., 2016) and images available in Allen brain atlas (http://www.brain-map.org/).

It is well established that GPR4 is a Gs-coupled receptor which drives the activity of adenylate cyclase (AC, (Ludwig et al., 2003), although signaling via G_13_ and G_q/11_ has also been documented (Tobo et al., 2007). However, the pH-sensitivity profile of GPR4-mediated cAMP accumulation may have a major effect on the role of this receptor in the body and brain specifically. It seems that this profile may be influenced by the methods used in different laboratories as it varies between the published reports. Using transiently transfected HEK293 cells (Ludwig et al., 2003), reported that GPR4 is inactive at pH > 8.0, highly active at physiological pH (7.4) but further acidification to pH 6.8 (which is a plausible range of physiological acidification) only results in a very small further activation of GPR4. In contrast (Tobo et al., 2007), reported significant activation of GPR4 in response to acidification from 7.4 to 7.0, which is within the range of physiological pH changes. At the same time, relatively little is known about pH-dependency of GPR4 in cells which express it naturally (such as endothelium).

Furthermore, protons which are detected by GPR4 are mainly derived from either carbonic acid or l-lactic acid (LL). Considering known signaling properties of LL (Lauritzen et al., 2013; Tang et al., 2014; Mosienko et al., 2015)we hypothesized that GPR4 could be additionally modulated by LL. This may have implications for various scenarios involving acidosis (respiratory *vs* metabolic).

In this study, we evaluated the CNS expression profile of GPR4 using a cell lineage tracing mouse model, which enables identification of cells where the GPR4 locus is activated during ontogeny. To verify that the expression persists in mature animals we employed a highly sensitive RNAscope in situ hybridization technology. We then compared the pH-activation profile of GPR4 expressed using a recombinant approach in HEK293 cells with that of human vascular endothelium cells (HUVEC) which natively express GPR4 and tested the hypothesis that GPR4 activity can be modulated by LL. Next, we studied the pharmacological properties and physiological effects of a novel GPR4 antagonist, NE 52-QQ57, developed by Novartis (compound 13 in (Velcicky et al., 2017)). This molecule is a centrally active GPR4 antagonist, effective after oral administration. Here, we confirm the high potency of NE 52-QQ57 as a GPR4 blocker and then evaluate its effects on central respiratory CO_2_ chemosensitivity in mice and rats.

## 2 Methods

### 2.1 Cloning of pCMV-GPR4-hIRES-EGFP

The GPR4 PCR product was obtained from rat genomic DNA and cloned between BglII and HindIII sites in pCMV-hIRES-EGFP plasmid to allow bi-cistronic expression of EGFP as a marker of expression. Sequence of the GPR4 insert was confirmed.

### 2.2 cAMP measurements in HEK293 cells transiently transfected with GPR4 and in HUVEC

The GloSensor assay was used for cAMP measurements as previously described, with modifications (Binkowski et al., 2011). Transfection was optimised for the different two cell lines. HEK293 cells were plated in 96-well white polystyrene plates (Greiner Bio-One) in DMEM media (Gibco) supplemented with 10% FBS and 1% Penicillin/Streptomycin (Pen/Strep), at a density of 4 × 10^5^ cells/ml to achieve ∼70% confluence for transfection. After 20 h, cells were transiently co-transfected with the GloSensor cAMP plasmid GLO22F and pCMV-GPR4-hIRES-EGFP using Trans-IT 293 (Mirus) according to the manufacturer's protocol. Transfection efficiency was confirmed by fluorescent visualisation of EGFP. HUVEC were plated in 96-well white polystyrene plates in endothelial cell growth medium (Promocell) supplemented with 1% Pen/Strep. 2 × 10^4^ cells were seeded per well. Since HUVEC are difficult to transduce using chemical transfectants, we generated an adenoviral vector for CMV-driven GLO22F expression and used it with multiplicity of infection of 75.

26 h after the transfection, cells were incubated with 850μM beetle luciferin potassium salt (Promega) at pH 7.4 for 2 h in the dark. Prior to adding drugs, media was changed to HBSS (Gibco) buffered with 20mM HEPES and titrated to the desired pH. Cells were incubated with NE 52-QQ57 for 20 min in a final well volume of 100μl. Luminescence measurements of cAMP accumulation were obtained using a Tecan microplate reader (Infinite M200 PRO).

LL solutions were titrated to neutral pH (7.4) using NaOH and added to the media, while resultant pH was carefully controlled. A water soluble forskolin analogue NKH 477 (Santa Cruz Biotechnology, 0.1–100μM) was used as a positive control to activate AC in a receptor-independent manner.

Non-transfected HEK293 cells were unresponsive to pH changes such as used in these experiments.

### 2.3 Assessment of the activity of NE 52-QQ57

10mg of NE 52-QQ57 was first dissolved in 300μl DMSO and then further diluted into HBSS as required. In order to assess the efficacy of NE 52-QQ57 in blocking GPR4-mediated cAMP accumulation, experiments were performed at a range of pH (8.0–6.8). For estimation of IC_50_ of NE 52-QQ57, tests were performed at pH 7.4.

### 2.4 Immunofluorescent localisation of GPR4 in the mouse brain

In order to investigate the pattern of GPR4 expression in the brain, we generated a lineage tracing mouse model by expressing CRE recombinase (CreN) from the GPR4 locus. Homologous recombination of the GPR4-CreN targeting vector was achieved by transfection of the targeting vector into BALB/c mouse embryonic stem (ES) cells. Correctly targeted ES cells were selected by PCR and Southern blot. Targeted BALB/c ES cells were injected into C57Bl/6 host blastocysts and breeding of chimeric mice resulted in GPR4-CreN mice. These mice were then crossed with a reporter mouse harboring a floxed-STOP-EGFP cassette inserted into the Rosa26 locus. Removal of the STOP cassette by CreN-mediated loxP-recombination enables reporter expression in cells where CreN was present. Description of the GPR4 expression in peripheral tissues in this animal model is outside of the scope of the present paper.

Male mice (postnatal day 15 or 30) were then deeply anaesthetised with isoflurane, and transcardially perfused with phosphate buffered saline (PBS), followed by 4% paraformaldehyde (PFA). Brains were removed and fixed in 4% PFA, and then transferred to 30% sucrose. For further storage, brains were kept in PBS containing 0.02% sodium azide. Sequential 40μm sections were cut using a freezing microtome. Prior to antibody staining sections were incubated with antigen retrieval reagent (Polysciences) following blocking solution containing 10% goat serum and 0.03% Triton X-100. Free-floating sections were stained with antibodies diluted in PBS containing 1% goat serum and 0.03% Triton X-100. Anti-GFP (Invitrogen) and anti-TH (Santa Cruz) primary antibodies were applied in concentration of 1:400 and 1:50 respectively. For immunofluorescence, Alexa-488 and Alexa-594 (Thermo Fisher Scientific) secondary antibodies were used as recommended by the manufacturer. Fluorescent sections were cover slipped in Vectorshield™, and GPR4 expression throughout the brain was assessed using a confocal microscope (Leica SP5).

### 2.5 Fluorescent in situ hybridization (RNAscope) of GPR4 transcript in the mouse brain

A nine week old C57BL/6J mouse was euthanized by pentobarbital and immediately transcardially perfused with PBS, followed by 4% PFA. The brain was postfixed in 4% PFA overnight at 8 °C, followed by cryoprotection in 20% Sucrose PBS at 8 °C for 24 h (all solutions RNAse free). Brain tissue was then embedded in O.C.T. matrix, and stored at −80 °C until sectioning. 15μm Coronal sections were cut using a cryostat and mounted on superfrost plus microscope slides. Mounted sections were used for fluorescence in situ hybridization (FISH) using RNAscope multiplex fluorescent assay (Advanced Cell Diagnostics) according to manufacturer instructions. Fluorescent sections were cover slipped in Invitrogen ProlongTM Gold antifade reagent (Thermo Fischer Scientific). GPR4 transcript localization was assessed using a confocal microscope (Leica SP5).

### 2.6 Evaluation of NE 52-QQ57 effects *in vivo*

All the experiments in the UK were performed in accordance with the European Commission Directive 2010/63/EU (European Convention for the Protection of Vertebrate Animals used for Experimental and Other Scientific Purposes) and the UK Home Office (Scientific Procedures) Act (1986) with project approval from the Institutional Animal Care and Use Committees. Animal handling at Novartis was performed in accordance with animal protocols approved by the Kantonales Veterinäramt Basel (License number 2586/2015).

#### 2.6.1 Whole body plethysmography in rats and mice

Respiratory rate (RR, breaths min^−1^) and tidal volume (V_T_, a.u) were measured by whole-body plethysmography in 10 male C57Bl/6J mice (Charles River, UK), 20–27g and 8 male Sprage-Dawley rats, 98–112g, (UCL Biological Services colony). In brief, animals were placed in a Plexiglas recording chamber (volume 400 ml for mice or 1l for rats) that was slightly pressurised by a continuous inflow of humidified room air (∼1 L min-1). All experiments were performed at room temperature (22–24 °C). The animals were allowed ∼15 min to acclimatise to the chamber environment at normoxia/normocapnia (21% O_2_, 79% N2, and <0.3% CO_2_). Normoxic hypercapnia was induced by switching to a pre-mixed gas containing 21% O_2_ with 5 or 10% CO_2_ and the balance made up by N_2_. The hypercapnic mixtures were applied for 5 min in succession. The measurements of RR and V_T_ were taken during the last 2 min before exposure to hypercapnia and the last 2 min of each hypercapnic episode, when breathing stabilised. Hypercapnia-induced changes in the RR, V_T_, and minute ventilation (V_E_) (RR × V_T_) were averaged and expressed as mean ± SEM. Each animal underwent recordings with vehicle (25% v/v DMSO in 0.9% Saline; 200μl per mouse or 1ml per rat, i.p) and drug (NE-5-0057; 20mg kg-1, i.p) which were injected immediately before introduction into the plethysmography chamber, thus allowing at least 15 min for the effect to develop.

#### 2.6.2 Surgical procedures in rats

Male Wistar rats, 8–9 weeks of age, were anaesthetised (induction 5% isoflurane, maintenance α-chloralose 75mg kg^−1^, i.v.) and instrumented for blood pressure recording via femoral artery cannulation. The femoral vein was cannulated for administration of anaesthetic. The depth of anaesthesia was monitored by the stability of blood pressure and heart rate. The trachea was cannulated low in the neck with a 12 gauge cannula to maintain airway patency and/or for mechanical ventilation. Arterial blood gases were monitored using a blood gas analyser (Model 380EX, Siemens, Dortmund, Germany) and maintained at *P*O_2_ ∼120 mmHg, *P*CO_2_ 35–40 mmHg, and pH at 7.35–7.45. Body temperature was maintained at 37.0 ± 0.5 °C. At the end of the experiments, the animals were humanely killed by an overdose of pentobarbitone sodium (200mg kg^−1^, i.v.).

#### 2.6.3 Evaluation of the effects of NE 52-QQ57 on neurovascular coupling in rats

The rat head was secured within the fMRI scanner with ear and incisor bars, after which mechanical ventilation was started with oxygen-enriched air using an MRI compatible ventilator (CWE). Neuromuscular blockade was induced by gallamine (10mg kg^−1^; i.v.) and supplemented as necessary. Imaging was performed using a 9.4T Agilent horizontal bore scanner (Agilent). A 72mm inner diameter volume coil was used for transmission (Rapid Biomedical) and signal was received using a 4-channel array head coil (Rapid Biomedical). A flow sensitive alternating inversion recovery (FAIR) arterial spin labelling (ASL) MRI sequence was applied with a single shot gradient-echo echo planar imaging readout for simultaneous capture of cerebral blood flow (CBF) and T2* weighted blood oxygen level dependent (BOLD) data as described previously (Wells et al., 2015). The following sequence parameters were used: TR = 5000ms, TI = 2000ms, matrix size = 64 × 64, FOV = 35mm × 35mm, TE = 10ms, single slice (slice thickness = 2mm), inversion pulse bandwidth = 20,000Hz (Christie et al., 2012). BOLD and CBF responses in the somatosensory cortex were triggered by electrical stimulation of the forepaw. FAIR images were continuously acquired for ∼6 min during a block design consisting of three periods of 60s “rest” followed by 60s forepaw stimulation (3Hz, 0.3ms pulse width, 1.5mA). Two baseline BOLD/CBF time-series measurements were acquired (∼12mins total imaging time). NE 52-QQ57 (10–20mg kg^−1^; i.p.) or vehicle (25% DMSO in phosphate buffered saline; 1.5ml kg^−1^) was then administered and two further BOLD/CBF time-series measurements were repeated 15 min after drug/vehicle administration. Vehicle injections were without any effects (data not shown).

*Data analysis: fMRI.* BOLD signals were taken from the “control” images following the global inversion pulse. CBF and BOLD time-series data, before and after drug/vehicle, were extracted from a manually drawn ROI in the unilateral forepaw region of the somatosensory cortex. The ASL CBF time-series data were corrected for contamination of BOLD effects at the time-points that coincide with the start and end of the forepaw stimulus where the BOLD signal is most different between tag and control conditions. For each animal, the average CBF/BOLD signal before and during forepaw stimulation was taken.

To generate spatial maps of significant BOLD signal changes to forepaw stimulation, BOLD images were spatially smoothed (0.5mm FWHM Gaussian kernel), and first level analysis of each time-series using an on/off regressor derived from the applied forepaw stimulus paradigm (and convolved with the standard HRF (SPM)) was applied to generate a statistical map before and after NE 52-QQ57/vehicle administration. In order to visualise the BOLD activation maps to forepaw stimulation, a minimum threshold of p < 0.05 with family wise error correction with a cluster size of >5 voxels was chosen.

### 2.7 Evaluation of cardiorespiratory effects of NE 52-QQ57 in rats

#### 2.7.1 Peripheral application

With animals spontaneously breathing, the tracheal cannula was connected to an open-circuit gas delivery system that allowed control of the composition of the inspired gas mixture. Two stainless steel braided wires (Advent RM, Oxford, UK) were implanted into the diaphragm to record diaphragmatic EMG. The signal was amplified (10,000x), filtered (500–1500Hz), rectified and smoothed (π = 50ms).

After a 15 min stabilization period following the preparative surgery, the animals were subjected to a hypercapnic challenge: CO_2_ concentration in the inspired gas mixture was increased to 10% for a period of 2 min. Following a 15 min recovery period, NE 52-QQ57 was administered (i.p.) at 10 min intervals to achieve cumulative doses of 5, 10 and 20mg kg^−1^ 10 min after the final dose was given, the hypercapnic challenge was repeated.

#### 2.7.2 Central application

Animals were transferred to a stereotaxic frame in the supine position. Mechanical ventilation was started using a positive pressure ventilator (Model 683; Harvard Apparatus) with a tidal volume of ∼2 ml and a ventilator rate similar to the normal respiratory frequency (∼60 strokes min^−1^). Neuromuscular blockade was induced by gallamine (10mg kg^−1^; i.v.) and supplemented as necessary (1–2mg kg h^−1^).

The ventral brainstem surface was exposed as described previously (Gourine et al., 2005). Briefly, superficial neck musculature was divided along the midline by blunt dissection and retracted with 3-0 sutures. The trachea and oesophagus were tied, bisected rostral to the tracheal cannula and reflected rostrally. The longus capitis muscle was partially excised to expose the occipital bone. Using a dental drill with a fine round-head bur, the bone was carefully removed leaving the dura intact. Upon completion of all surgical preparation the dura was incised and reflected laterally to expose the ventral surface of the brainstem. Constant flow of CSF covered the brain surface and was wicked away with a small piece of tissue. Peripheral chemodenervation was performed, with both vagi and carotid sinus nerves sectioned bilaterally. Activity from the central cut end of the right phrenic nerve was recorded as an indicator of central respiratory drive. The signal was amplified (20,000x), filtered (500–1500Hz), rectified and smoothed (π = 50ms).

After a 15 min stabilization period, the animals were subjected to a hypercapnic challenge; CO_2_ concentration in the inspired gas mixture was increased to 10% for 2 min. Following a 15 min recovery period, the vehicle (10% DMSO in artificial cerebro-spinal fluid) was applied directly to the exposed ventral surface of the brainstem. A second hypercapnic challenge was applied after 10 min. This sequence was repeated following the topical application of NE 52-QQ57 (1mM) on the ventral surface of the brainstem.

#### 2.7.3 Data analysis

Physiological variables were digitized using a Power 1401 interface (CED) and stored on a PC for offline processing using Spike 2 software (CED). Hypercapnia-induced changes in the respiratory rate, amplitude of phrenic nerve discharge or diaphragmatic EMG bursts, and neural minute ventilation (respiratory rate x burst amplitude) in control conditions, following application of the vehicle or GPR4 antagonist were compared with 2-way ANOVA. *P* values of <0.05 were considered significant. Hemodynamic variables were averaged over 60 s periods for 5 min after application of each dose of the GPR4 antagonist. The effects of GPR4 application on HR and BP were compared to a control period taken 5 min immediately preceding the injection of the first dose. An extra time point was taken 10 min after the application of the largest dose of NE 52-QQ57.

## 3 Results

### 3.1 GPR4 expression in the mouse brain

#### 3.1.1 Immunofluorescence in transgenic mice

**Fig. 1:**
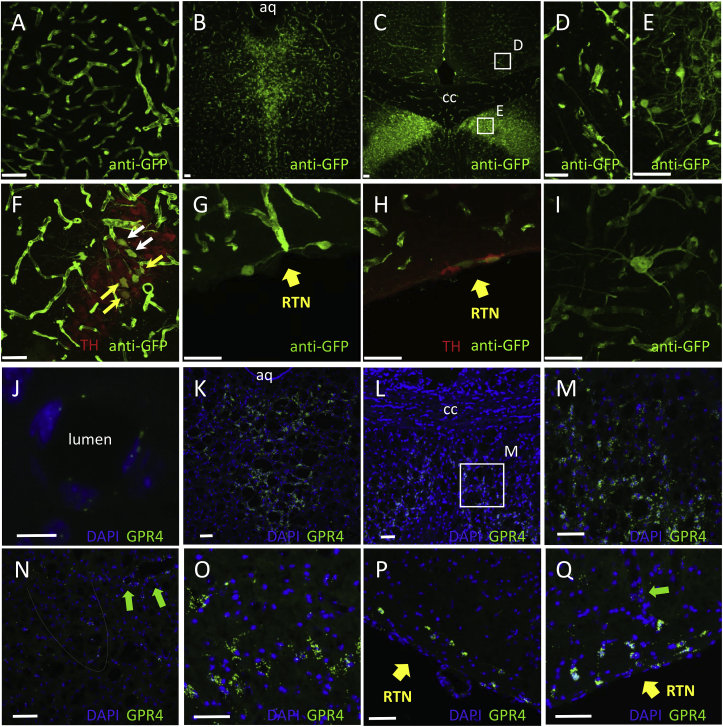
**GPR4 expression in the mouse brain**. **A-I.** Fluorescent immunohistochemistry of GPR4-CRE-STOP-EGFP mouse brain slices using antibodies against EGFP (anti-GFP) and TH (marker of catecholaminergic neurones). Unless indicated otherwise, scale bars are 50μm. **A.** GPR4 expression in blood vessels was prominent throughout the whole brain (see all panels). **B.** Strong and wide-spread GPR4 expression in dorsal raphe nucleus neurones.**C.** GPR4 expression in neurones of the lateral septum. **D, E.** Higher magnification images from areas indicated in panel C. **F.** In the locus coeruleus (A6 cell group) GPR4 expression was detected in neurones, some of which were TH-positive (yellow arrows) or TH-negative (white arrows). **G.** Ventral edge of the medulla oblongata at the level of RTN. A few RTN neurones could be detected in some sections. Less than 10 EGFP-positive cells per animal were found. **H.** Some of the sections at RTN level were double-stained for TH, but TH-positive cells were EGFP-negative and probably belonged to the C1 cell group. **I.** Scattered neurones of unclear phenotype were sporadically detected throughout the whole brain. **J-Q** – Results of RNAscope FISH. **J.** A blood vessel, scale bar 10μm **K.** Dorsal raphe **L.** lateral septum **M.** Lateral septum, magnified from L, scale bar 10μm **N.** Locus cortuleus, boundaries highlighted by the dotted line **O.** C1 cell group **P** and **Q**: RTN Green arrows in N and Q – blood vessels *aq – aqueduct; CC* – corpus collosum; *RTN* – retrotrapezoid nucleus See Supplement for the large scale versions of some of the images.

Mice in which CRE was knocked into the GPR4 locus were crossed with a ROSA-EGFP-reporter line to activate the dormant EGFP expression cassette. ROSA-reporter mice are considered to be the best model for ubiquitous expression in all cell types upon activation and have been extensively used for that purpose (Abe et al., 2013; Srinivas et al., 2001). Expression of the activate reporter EGFP was analysed throughout the whole brain using anti-GFP immunohistochemistry. It is acknowledged that the activation of the dormant EGFP transgene could occur at different stages of animal development, including the embryonic phase. Immunohistochemistry revealed very strong marker expression in cerebral blood vessels of all calibres with signal originating from the endothelial cells (Fig. 1A). The most prominent expression in non-vascular cells was detected in the dorsal raphe nucleus (Fig. 1B) and lateral septum (Fig. 1C-E). EGFP expression was also evident in area A6 (locus coeruleus), however, only a few EGFP-expressing neurones were found to be TH-positive, i.e. noradrenergic (Fig. 1F). Marker expression was undetectable in the C1 adrenergic cell group. Neuronal labelling was also detected in the RTN as reported previously (Kumar et al., 2015) (Fig. 1G and H). As expected, GPR4-positive neurones in the RTN were TH-negative (Fig. 1H). However, these neurones were scarce and had much lower level of marker expression compared to the neighbouring endothelial cells or raphe nucleus neurones (Compare Fig. 1 panels G,H with B and C).

Apart from these distinct nuclei, isolated EGFP-positive neurones were detected sporadically across the whole brain. These neurones were fairly large (20–35μm in diameter) with long and extensive processes but could not be identified as any known phenotype (Fig. 1I).

#### 3.1.2 RNAscope FISH

RNAscope labelling results in punctate staining which is likely to reflect local foci of mRNA in the cells which express the relevant gene (Fig. 1J-Q, see also high power images in the Supplement). Green puncta were detected in the same areas as in the knock-in mouse indicating that in a postnatal mouse neuronal expression persists in multiple locations, including RTN, raphe and lateral septum. Even though localisation of endothelium was rather difficult in the absence of an additional endothelial stain, on a number of images vascular structures were also clearly seen (green arrows in panels J,N, Q). Therefore, distribution of GPR4 revealed by FISH is qualitatively similar to that observed in the knock-in mouse.

### 3.2 Comparison of GPR4-mediated cAMP accumulation in transiently transfected HEK293 cells and in HUVEC which express GPR4 natively

**Fig. 2:**
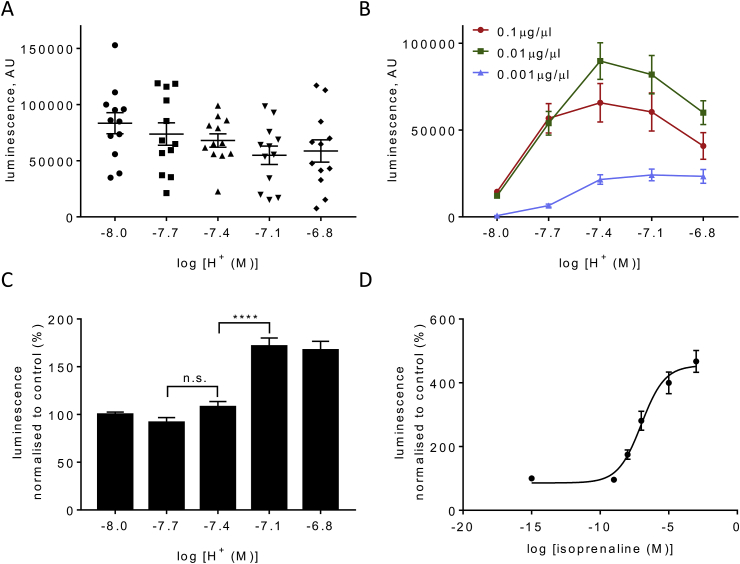
***In vitro* characterisation of GPR4 activity**. **A**. Increases in cAMP induced by a water-soluble forskolin analogue NKH 477 and detected using Glosensor assay are stable across the physiologically relevant pH range. 20μM NKH 477 was applied to naïve HEK293 cells (not GPR4 transfected). Data represent mean ± s.e.m (n = 4 of triplicates). Differences are not statistically significant (repeated measures one-way ANOVA). **B.** pH-dependent cAMP accumulation in GPR4 expressing HEK293 cells depends on the quantity of DNA used for transfection and by implication on the level of the expression of GPR4. Data represent mean ± s.e.m. for averages of triplicates (n = 7 of triplicates for 0.1 μg/μl, n = 3 of triplicates for 0.01 and 0.001 μg/μl). High levels of expression shifts the activation curve towards the alkaline range. Activation profile with 0.001 μg/μl DNA leads a profile which resembles response of HUVEC (see panel 2C) which express GPR4 natively. **C.** cAMP accumulation in HUVEC at different pH. Increase in cAMP without NE 52-QQ57 at pH 7.1 and 6.8 is statistically significant (n = 6 of triplicates, repeated measures one-way ANOVA shows p < 0.01) **D**. Concentration-response curve for cAMP production evoked using non-selective β-adrenoceptor agonist isoprenaline to illustrate that HUVEC cells can mount a robust cAMP response to a different Gs-coupled GPCR. Isoprenaline activates natively expressed β2 adrenoceptors in a concentration-dependent manner (n = 3 of triplicates). -∞ indicates zero concentration of the drug.

We initially verified that receptor-independent cAMP production triggered by the soluble forskolin analogue NKH 477 is pH independent within the physiologically relevant range (pH 8.0–6.8). As shown in Fig. 2A, 20 μM NKH 477 evoked similar effects across this pH range indicating that the assay was stable across the pH range, and that other factors, for example AC or phosphodiesterase activities, did not change significantly. Cell lines transfected with plasmids bearing strong promoters express transgenes at high level untypical for GPCR. Naïve HEK 293 cells do not respond to acidification with changes in cAMP (data not shown) consistent with the lack of GPR4 expression in this cell line (Atwood et al., 2011). We first expressed recombinant GPR4 from the rat using plasmids with a CMV promoter and found that acidification resulted in a robust increase in cAMP level (Fig. 2B). Strong activation occurred in the range between pH 8.0 and 7.4, while further acidification resulted in no further increase in cAMP levels. This profile closely resembles the one in the original report by (Ludwig et al., 2003) and suggests that GPR4 acts as a constitutive driver of cAMP production at neutral pH, but makes it hardly suitable for detection of physiological pH changes (7.4–7.0). Of note, the level of cAMP accumulation evoked by GPR4 at neutral pH is comparable to that achievable using 20 μM of NHK 477 (data not shown). We hypothesised that such a shift of pH sensitivity towards the alkaline range is a result of the high level of GPR4 expression which is common when using transient expression and plasmids with a strong promoter. We therefore tested pH sensitivity of GPR4-expressing HEK 293 cells using lower concentrations of DNA. As suspected, using 100 times less DNA shifted pH sensitivity towards more acidic values, closer to the physiological range (Fig. 2B).

However, it is extremely difficult to tune the level of the recombinant receptor expression to match the physiological levels. Therefore, we next used HUVEC, which express GPR4 natively and found that in these cells activation occurs mainly between pH 7.7 and 7.1 (Fig. 2C; see Supplement for additional statistical information for this and other datasets) which is closer to the feasible physiological range. At the same time, the potency of this signalling system in HUVEC appeared to be rather modest, since between pH 7.4 and 7.1 cAMP level only increased by ∼50%. For reference, activation of Gs-coupled β-adrenoceptors with isoprenaline led to >400% increase in cAMP (Fig. 2D).

### 3.3 NE 52-QQ57 effect on GPR4 in HEK 293 cells is pH dependent

**Fig. 3:**
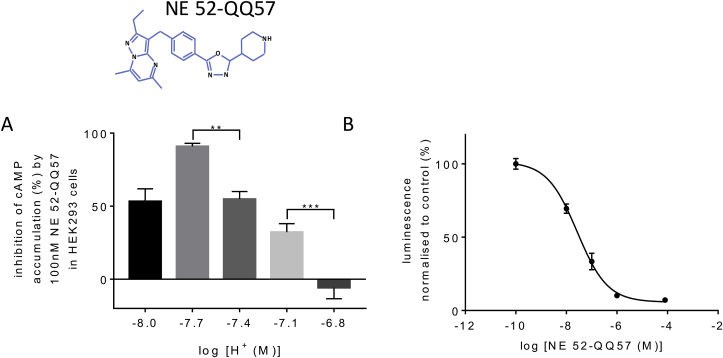
**NE 52-QQ57 is a novel GPR4 antagonist working in a physiological range of pH**. **A.** NE 52-QQ57 (formula above the bar chart) inhibits GPR4-mediated cAMP accumulation but its potency depends on proton concentration (pH). Plotted are mean % changes ± s.e.m. (n = 5 of triplicates) where % change is calculated as *(with drug – baseline)/(baseline x 100)* for every triplicate. Inhibition by NE 52-QQ57 becomes less effective as pH decreases. Significant differences are shown by repeated measures one-way ANOVA with Tukey's multiple comparisons post hoc test (** - p < 0.01). **B.** Concentration-response curve for NE 52-QQ57 at pH 7.4. Calculated IC_50_ is 26.8 nM. Data represent mean ± s.e.m. of triplicates. Log −10 corresponds to zero concentration of the drug.

The effect of NE 52-QQ57 on proton-mediated cAMP accumulation in cells over-expressing GPR4 was dependent on pH (Fig. 3A) and this dependence in the acidic range could be explained by competition with protons. NE 52-QQ57 (100nM) was maximally effective at pH 7.7 but approximately equally effective at pH 8.0 and 7.4. At pH 7.1, the potency of NE 52-QQ57 was reduced and at pH 6.8 NE 52-QQ57 (100nM) was ineffective. At pH 7.4, NE 52-QQ57 acts as a highly potent antagonist of GPR4 mediated cAMP accumulation with IC_50_ of 26.8nM (Fig. 3B).

### 3.4 LL inhibits GPR4-mediated pH dependent cAMP accumulation in HEK293 and HUVEC

**Fig. 4:**
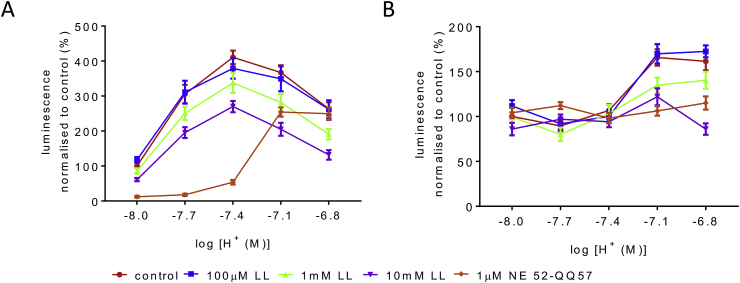
**LL inhibits cAMP accumulation induced by acidosis in a concentration-dependent manner in (A) HEK293 and (B) HUVEC cells**. All values are normalised to control at pH 8.0 for each triplicate measurement. Two-way repeated measures ANOVA revealed a significant effect of pH (p < 0.0001) and LL (p < 0.05) on cAMP levels in HEK293 cells. Post-hoc Sidak's multiple comparisons test shows confirmed significant differences between effects of 0 and 10mM LL (p < 0.05 at pH 7.4 and 6.8, p < 0.01 at pH 7.1) and between 100 μM and 10 mM (p < 0.05), but the difference between 1 and 10mM LL was not significant (p = 0.53 at pH 7.1, p = 0.78 at pH 6.8). In HUVECs, the same analysis revealed a significant effect of pH (p < 0.0001) and LL (p < 0.05) and a significant interaction effect (p < 0.001). The post-hoc test shows significant differences between 0 and 10mM (p < 0.05 at pH 7.1, p < 0.0001 at pH 6.8), between 100 μM and 10 mM (p < 0.05 at pH 7.1, p < 0.0001 at pH 6.8), and between 100 μM and 1 mM (p < 0.05 at pH 6.8).n = 4–5 of triplicates for each data point in (A) and (B), apart from n = 3 for 1μM NE 52-QQ57 curve in HUVEC. 1μM NE 52-QQ57 antagonises pH dependent activation of GPR4, the block is overcome at acidic pH in HEK293 cells.

When LL was applied to GPR4-transfected HEK293 cells (DNA concentration 0.1 μg/μl) or to HUVECs at a range of pH values, it concentration-dependently inhibited the cAMP accumulation induced by acidosis (Fig. 4A and B). In HEK293 cells the effect was significant at 10mM LL, but in HUVEC, 1 and 10 of LL significantly inhibited the pH responsiveness (Fig. 4B). NE 52-QQ57 (1 μM) antagonised GPR4-mediated responses in HEK293 cells but its efficacy was reduced at acidic pH (7.1 and 6.8) presumably due to high level of activation of the over-expressed receptor (Fig. 4A). In HUVECs, 1 μM of NE 52-QQ78 completely blocked GPR4-mediated cAMP responses at all pH levels tested (Fig. 4B).

**Fig. 5:**
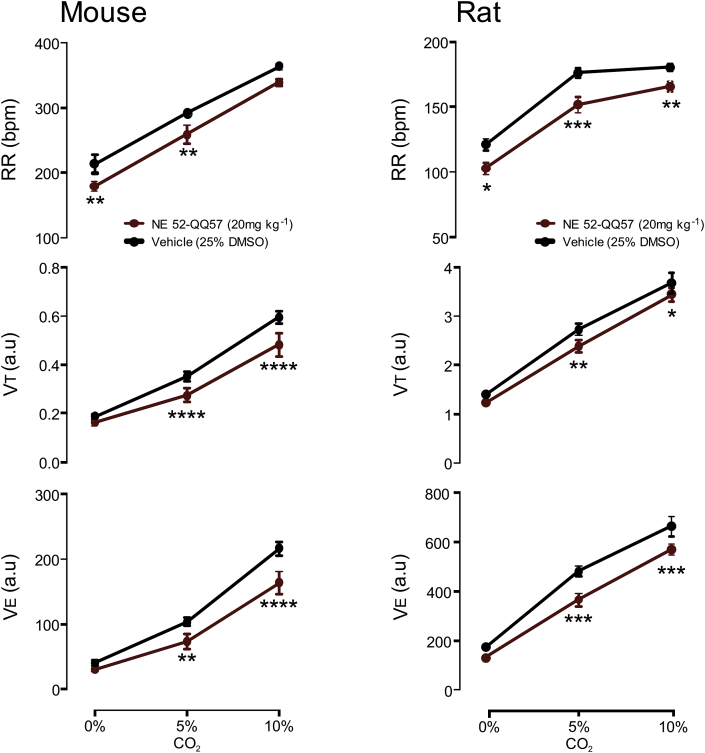
**NE 52-QQ57 blunts hypercapnic response to 5% and 10% CO2 in unanaesthetised mice (n**=**10) and rats (n**=**8)**. V_T_ and V_E_ measured at 5 and 10% CO_2_ were significantly reduced by NE 52-QQ57 (20 mg kg^−1^ i.p.) relative to the vehicle group (25% DMSO).**p < 0.01 and ****p < 0.0001 vs. vehicle, repeated measurement two-way ANOVA followed by Sidak's multiple comparisons test.

### 3.5 Central effects of NE 52-QQ57 on CO_2_-induced respiratory responses in unanaesthetised mice and rats

Minute ventilation (V_E_) which is the cumulative of RR and V_T_ was not significantly affected at baseline by 20 mg kg^−1^ of NE-52-QQ57 in either rats (n = 8) or mice (n = 10). Relative to the vehicle (25% DMSO), NE-52-QQ57 significantly attenuated V_E_ during hypercapnia at 5 and 10% CO2 in both species. For example, with 10% CO_2_ V_E_ increased to 216 ± 11 a.u. in the group treated with vehicle, but only to 164 ± 18 a.u. in mice treated with NE-52-QQ57 (p < 0.001). Similarly, in rats at 10% CO_2_ V_E_ increased to 662 ± 40 a.u. in the vehicle group, but to 568 ± 22 a.u. in the treated group (p < 0.001). Thus, in freely behaving mice and rats NE22-QQ57 blunted hypercapnic response to CO_2_.

### 3.6 Evaluation of NE 52-QQ57 cardio-respiratory effects in anaesthetised rats

**Fig. 6:**
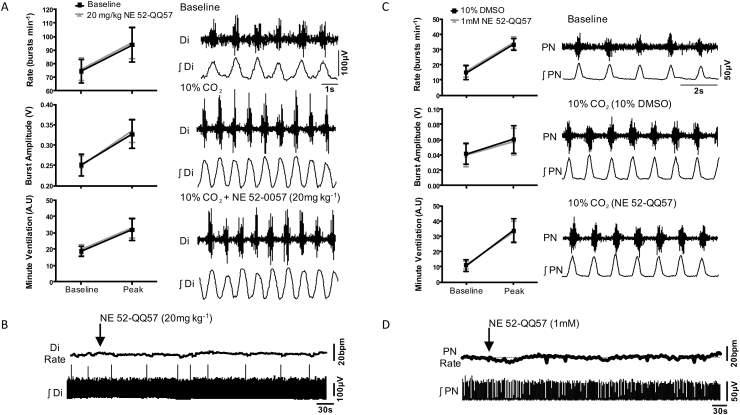
**Peripheral or central administration of NE 52-QQ57 has no effect on CO**_**2**_**-induced respiratory responses**. **A.** Averaged responses (left) and raw traces (right) to illustrate responses to hypercapnia after i.p. administration of NE 52-QQ57 (20 mg kg^−1^). Di – raw activity of the diaphragm, ʃDi – integrated activity of the diaphragm to illustrate respiration. **B.** Effect of i.p. administration of NE 52-QQ57 (20 mg kg^−1^) on baseline diaphragmatic EMG activity. **C.** Responses to hypercapnia after topical application of NE 52-QQ57 (1mM) on the ventral surface of the medulla oblongata. NE 52-QQ57 had no effect on CO_2_-induced responses (two-way ANOVA). PN – activity of the phrenic nerve. **D.** Effect of direct application of NE 52-QQ57 (1mM) on the ventral surface of the medulla oblongata on baseline phrenic nerve activity. PN Rate – frequency of respiration measured from the ʃPN traces.

In anaesthetised rats, hypercapnia (10% CO_2_ in the inspired air) increased respiratory rate from 77 ± 9 to 94 ± 13 bursts/min (Fig. 6A), and this was not affected following systemic administration of NE 52-QQ57 (20 mg kg^−1^; n = 8, 76 ± 9 to 95 ± 12 bursts min^−1^; p = 0.92). NE 52-QQ57 had no effect on CO_2_-evoked increases in diaphragm EMG amplitude (0.25 ± 0.03 to 0.33 ± 0.04V cf. 0.25 ± 0.02 to 0.34 ± 0.03V; p = 0.94) and minute ventilation (19 ± 3 to 32±7A.U cf. 20 ± 3 to 33±6A.U; p = 0.88). Systemic NE 52-QQ57 also had no effect on basal respiratory rate (Fig. 6B). To additionally ensure that NE 52-QQ57 reaches the central GPR4 targets, the drug was then delivered directly on the ventral surface of the brainstem, where the RTN is located. Hypercapnia (10% CO_2_ in the inspired air) in 7 animals with denervated peripheral chemoreceptors increased respiratory frequency, phrenic nerve amplitude and minute ventilation (Fig. 6C). Hypercapnia increased the respiratory rate from 18 ± 6 to 40 ± 5 bursts min^−1^ following application of the vehicle on the ventral brainstem surface. CO_2_ had a similar effect in the presence of NE-52-QQ57 (1 mM) on the ventral brainstem surface (18 ± 4 to 41 ± 5 bursts min^−1^; p = 0.86, Fig. 6C). NE 52-QQ57 had no effect on other components of the hypercapnic respiratory response including increases in phrenic nerve burst amplitude (0.04 ± 0.01 to 0.06 ± 0.02V vs. 0.04 ± 0.02 to 0.06 ± 0.02V; p = 0.92) and minute ventilation (11 ± 4 to 34±8A.U vs. 11 ± 2 to 33±7A.U; p = 0.97). Direct application of NE 52-QQ57 to the ventral medulla had no effect on baseline respiratory activity (Fig. 6D).

### 3.7 Evaluation of the NE 52-QQ57 effects on hemodynamics and neurovascular coupling

Systemic administration of NE 52-QQ57 (up to 20 mg kg^−1^ i.p., n = 7) did not affect heart rate and blood pressure in anaesthetised rats (Supplementary Fig. 1). Neither did the drug affect CBF and BOLD response to somatosensory stimulation (electrical stimulation of the forepaw). There were no differences in the spatial extent and magnitude of the evoked BOLD signals.

## 4 Discussion

We have investigated the signalling properties, CNS distribution and physiological role of GPR4 in the mechanisms of cardiorespiratory control. Previous work had established that, in peripheral tissues, GPR4 is prominently expressed in the endothelium of blood vessels (Huang et al., 2007; Kim et al., 2005; Qiao et al., 2006; Wyder et al., 2011), where its deletion can cause major abnormalities (Yang et al., 2007). In the brain, presence of GPR4 was confirmed for vascular endothelium (Sun et al., 2016). In addition, transcriptome data unequivocally map GPR4 to endothelium with hardly any expression in other brain cell types, at least in front brain regions (Zhang et al., 2014, 2016). Surprisingly, a recent study reported no GPR4 expression in the CNS blood vessels using in situ hybridisation. Instead single cell PCR detected GPR4 in neurones of a few key brainstem nuclei including the RTN (91% GPR4-positive neurones), catecholaminergic area C1 (83%) and serotonergic raphe nucleus (100%) (Kumar et al., 2015). Here we employed a GPR4-CRE reporter knock-in mouse where expression of CRE is driven by the endogenous locus of the GPR4 gene and leads to the expression of CRE in all cells in which GPR4 is expressed during ontogeny, allowing cell lineage tracing by crossing these mice with a reporter line where expression of EGFP can be induced by CRE. Given that CRE is driven by the endogenous locus of the GPR4, it is assumed that it obeys strictly the same rules as the actual transcript encoded by this locus. We found that the EGFP was highly and abundantly expressed in all types of cerebral blood vessels (Fig. 1A and B,C,F,G,H). Labelled vascular cells were found to be mainly (if not exclusively) endotheliocytes, because the expression can be traced all the way down to the capillary level and up the walls of the larger vessels (arterioles and veins). In addition, prominent EGFP expression was also found in neurones of the dorsal raphe nuclei (Fig. 1B) this is consistent with the serotonergic phenotype expressing GPR4 according to (Kumar et al., 2015). Another area of strikingly high marker expression is the lateral septum (Fig. 1C) which was never reported before. The GPR4-CRE reporter approach leaves room for potential overestimation of the spread of the gene of interest because expression of GPR4-CRE and CRE-dependent activation of the reporter gene may occur during early stages of development, while in the adult the GPR4 locus could be repressed in some of these cells. Therefore, we employed the recently introduced FISH technique and have obtained comparable results. RNAscope detection is extremely sensitive and the staining appears as puncta localised within the cell, rather than an evenly spread fluorescence (Fig. 1J-Q). FISH confirmed GPR4 expression by various neuronal populations around the brain, including RTN, raphe, A6 group (locus coeruleus) and lateral septum. Since these results were obtained from a mature mouse, they confirm that our findings in the knock-in model were not a result of embryonic activation of the GPR4 locus. Expression in endothelium could also be detected, although without counter-staining (which we could not perform), the blood vessels could only be identified by their shape. Admittedly, the density of FISH signal in the endothelium was less than we expected from the lineage tracing study, suggesting some reduction in GPR4 level in the endothelium in the mature animals. Interestingly, both, raphe nucleus (Balaban, 2002; Deakin, 1991; Graeff et al., 1996; Mosienko et al., 2012) and lateral septum (Albert and Walsh, 1982; Talishinsky and Rosen, 2012; Yadin et al., 1993) have been extensively implicated in control of anxiety and aggression. Our study justifies further exploration of potential role of GPR4 in these nuclei if GPR4 protein presence in them in adult animals can be confirmed at protein level. It also points to the very likely role of GPR4 in regulation of brain endothelial function and signalling between endothelium and parenchyma which we postulated earlier (Paton et al., 2002).

The pioneering study which for the first time established GPR4 as a proton sensitive receptor (Ludwig et al., 2003)reported that the intracellular coupling for this receptor occurs via AC and that its activation occurs mainly between alkaline (∼8.0) and neutral (∼7.3) pH. This “flat” profile in the physiological pH range (7.4–7.0) was also documented by (Tobo et al., 2007) making it difficult to envisage this receptor as a primary pH sensor in the brainstem, where pH is rather tightly controlled and kept within ∼7.6–7.0. In this study we re-evaluated GPR4-mediated proton-activated cAMP production in two model cell systems: HEK293 cells transiently transfected to overexpress GPR4, and HUVEC cells which express GPR4 natively. We first confirmed that the assay is sufficiently stable across the relevant pH range, this being necessary in light of previous reports of pH sensitive species of AC (Anand-Srivastava and Johnson, 1980; Birnbaumer and Yang, 1974). In HEK293 cells, transfected with recombinant GPR4 (but not naïve HEK293), cAMP accumulation occurred in the alkaline range, between pH 8.0 and 7.4 (Fig. 2B) with no further cAMP increase at acidic pH (6.8). This operational range would be hardly suitable for CO_2_ monitoring in the brain. In contrast, HUVEC cells had a radically different activation profile (Fig. 2C) whereby essentially all GPR4-mediated cAMP accumulation occurred between pH 7.4 and 7.1. This range compares well to previous findings in HUVEC (Wyder et al., 2011). We hypothesised that the alkaline-shifted operational range of GPR4 in HEK293 cells registered here and in several previous studies could be explained by the high receptor expression levels typical for transient expression experiments in cell lines. Indeed, by lowering concentration of the GPR4 expressing plasmid we obtained a profile which better resembled the activation curve in HUVEC (Fig. 2B and C).

**Fig. 7:**
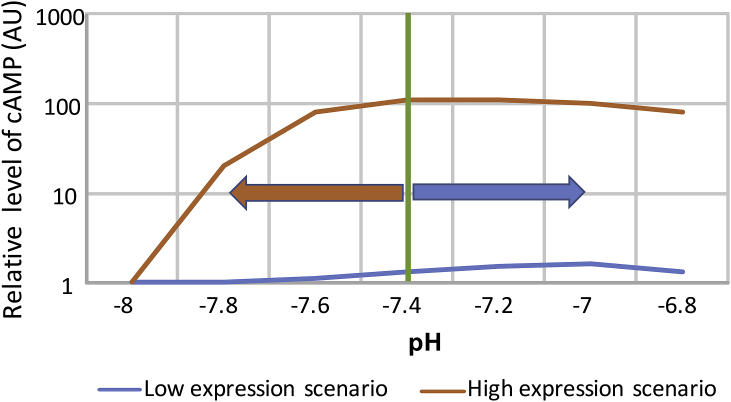
**Schematic to illustrate the putative impact of GPR4 expression level on its function**. High level of expression (as exemplified by transiently transfected HEK293 cells) leads to production of copious amounts of cAMP at neutral pH (7.4) and makes the system insensitive to physiological acidification. Low, natural level of expression, such as in HUVEC, reveals GPR4 mediated responses within the physiological window (7.4–6.8) but limits the potency of GPR4-mediated signaling via cAMP, making it unsuitable for fast and robust cellular responses.

Therefore, as illustrated in Fig. 7, relatively high GPR4 levels (as in overexpressing HEK293 cells) are likely to lead to high GPR4 activity at neutral pH 7.4. In the case of a neurone, this could make it more excitable and active at neutral pH. At the same time, such an activation profile would be unsuitable for the detection of the changes in pH within physiologically relevant range (e.g. 7.4 to 6.8). Lower level of expression (as exemplified by HUVEC) offers a mechanism for the detection of physiological acidification but the potency of this mechanism seems to be limited. cAMP in HUVEC only increased by ∼50% (Fig. 2C), a rather modest effect when compared to that of a canonical activator of Gs signalling, the β2 adrenoceptor (Fig. 2D). Possibly, cAMP-independent signalling may be more important for the action of GPR4 (see introduction), this includes also slow, genomic mechanisms which only become relevant under chronic conditions, such as during tumour formation.

Protons which can activate GPR4 are mainly derived from carbonic acid and LL, the two key tissue acids. Concentrations of LL in plasma and brain are highly dynamic, in most reports ranging between 100 μM and 5 mM, and increasing to 10 mM under conditions of hypoxia, metabolic stress or severe exercise (Mosienko et al., 2015). We found that LL has, irrespective of the action of protons, an additional effect on GPR4. Both in HEK293 and HUVEC, LL reduced proton-mediated cAMP accumulation mediated by GPR4 (Fig. 4). This effect was evident at 1 mM and increased significantly with 10 mM of LL. We cannot completely exclude the possibility that high concentrations of LL (e.g. 10 mM and above) might have effects unrelated to the signalling mechanism under study, for example via LL effects on energy production and/or mitochondrial redox state. Nevertheless, the data obtained suggest that LL at biologically relevant concentrations acts as a negative (possibly allosteric) modulator of GPR4-mediated signalling. The data also suggest that under physiological blood and brain LL concentration (usually ∼1 mM), GPR4 is partially tonically inhibited by LL and its sensitivity to protons is lower than could be expected from *in vitro* assays.

The recently published GPR4 blocker, NE 52-QQ57 (Velcicky et al., 2017), represents a promising new family of drugs, because GPR4 is one of the potential targets in cancer therapy (Castellone et al., 2011; Damaghi et al., 2013) and inflammatory responses (Chen et al., 2011; Dong et al., 2013). Our experiments confirm NE 52-QQ57 as a highly potent but pH-dependent inhibitor of GPR4-mediated cAMP accumulation with an IC_50_ of 26.8 nM at physiological pH (7.4) in HEK293 cells. It was less effective under acidic (pH 7.1–6.8) or alkaline (pH 8) conditions but 1 μM of NE 52-QQ57 completely abrogated any pH dependent cAMP responses in HUVEC. However, clinical application of GPR4 blockers would be problematic if these drugs had serious adverse effects on cardio-vascular and/or respiratory homeostasis.

We investigated this issue in awake mice and rats. In unanaesthetised animals 20 mg kg^−1^ of NE 52-QQ57 caused a small, but significant reduction in the ventilatory response to hypercapnia evoked by 5 and 10% CO_2_. This effect is consistent with the phenotype of GPR4 knock-out mice (Kumar et al., 2015). With reference to the *in vitro* parts of our study, the findings in mice may be best described by the “low level of expression” scenario (Fig. 7), whereby GPR4 acts as a weak constitutive driver of cAMP with a small additional capacity to further elevate cAMP during physiological acidification. In anaesthetised rats, systemic peripheral administration of NE 52-QQ57 in doses of up to 20 mg kg^−1^ had no effect on respiratory CO_2_ chemosensitivity (Fig. 5), resting arterial blood pressure and heart rate. Direct application of NE 52-QQ57 onto the ventral surface of the brainstem also did not affect cardio-respiratory homeostasis (Fig. 6). Neurovascular coupling in the somatosensory cortex (Supplementary Fig. 1) was also fully preserved.

One possible reason for the lack of NE 52-QQ57 effect on CO_2_ sensitivity under anesthesia is the inhibitory action of LL on GPR4 as demonstrated here (Fig. 4). Indeed, some anaesthetics such as urethane can significantly increase LL concentration (Alfaro and Palacios, 1997), although we were unable to find similar evidence for chloralose which was used here. If, however LL is increased by the anaesthetic, it could mask the GPR4-mediated effects. At the same time, we know that the other mechanism of chemosensitivity which resides in astrocytes is fully functional under chloralose anaesthesia (Gourine et al., 2010).

Altogether, NE 52-QQ57 has no serious detrimental effects on cardiovascular and respiratory systems in rodents, and this may be considered as encouraging news for further development of potential clinical applications of GPR4 antagonists. The recent demonstration of antinociceptive activity of NE 52-QQ57 highlights a potential avenue for exploration (Velcicky et al., 2017), as does the potential application of another GPR4 antagonist for treatment of myocardial infarction (Fukuda et al., 2016). Further promising applications involve cancer therapy (Justus et al., 2013; Wyder et al., 2011; Yang et al., 2007), neuro-inflammation, or renal disease. The biological significance of GPR4 expression in multiple neuronal populations requires further investigation, especially given the association of some of these nuclei with anxiety and responses to stress.

## Financial support

This work was supported by the grants from BBSRC: BB/L019396/1, BB/K009192/1; MRC MR/L020661/1; BHF RG/14/4/30736; Wellcome Trust 095064.

## Conflicts of interest

The authors declare no conflict of interest.
