# Supplementary material for: CNS distribution, signalling properties and central effects of G-protein coupled receptor 4
Source: Neuropharmacology. 2018 Aug;138:381–92. doi: 10.1016/j.neuropharm.2018.06.007 (PMC6063991; doi:10.1016/j.neuropharm.2018.06.007)
Supplement: Multimedia component 1 [file mmc1.pdf]

Supplementary table 1. Statistical analysis of different experimental conditions related to Figure 2C.

Tukey's multiple comparisons test	Summary	Adjusted P Value
-8.0 vs. -7.7	ns	0.8988
-8.0 vs. -7.4	ns	0.9162
-8.0 vs. -7.1	****	<0.0001
-8.0 vs. -6.8	****	<0.0001
-7.7 vs. -7.4	ns	0.4177
-7.7 vs. -7.1	****	<0.0001
-7.7 vs. -6.8	****	<0.0001
-7.4 vs. -7.1	****	<0.0001
-7.4 vs. -6.8	****	<0.0001
-7.1 vs. -6.8	ns	0.9926

Supplementary table 2. Statistical analysis of different experimental conditions related to Figure 3A

Tukey's multiple comparisons test	Summary	Adjusted P Value
-8.0 vs. -7.7	***	0.0007
-8.0 vs. -7.4	ns	0.9998
-8.0 vs. -7.1	ns	0.1379
-8.0 vs. -6.8	****	<0.0001
-7.7 vs. -7.4	**	0.0012
-7.7 vs. -7.1	****	<0.0001
-7.7 vs. -6.8	****	<0.0001
-7.4 vs. -7.1	ns	0.0948
-7.4 vs. -6.8	****	<0.0001
-7.1 vs. -6.8	***	0.0006

Supplementary table 3. Statistical analysis of different experimental conditions related to Figure 4A.

Dunnett's multiple comparisons test	Summary	Adjusted P Value
**-8.0**		
control vs. 100µM LL	ns	0.8724
control vs. 1mM LL	ns	0.9281
control vs. 10mM LL	ns	0.3477
**-7.7**		
control vs. 100µM LL	ns	0.9957
control vs. 1mM LL	ns	0.1477
control vs. 10mM LL	***	0.0002
-7.4		
control vs. 100µM LL	ns	0.5618
control vs. 1mM LL	*	0.0342
control vs. 10mM LL	****	0.0001
-7.1		
control vs. 100µM LL	ns	0.8677
control vs. 1mM LL	**	0.0097
control vs. 10mM LL	****	0.0001
-6.8		
control vs. 100µM LL	ns	0.9999
control vs. 1mM LL	*	0.0358
control vs. 10mM LL	****	0.0001

Supplementary table 4. Statistical analysis of different experimental conditions related to Figure 4B.

Dunnett's multiple comparisons test	Summary	Adjusted P Value
**-8.0**		
control vs. 100µM LL	ns	0.5452
control vs. 1mM LL	ns	0.9999
control vs. 10mM LL	ns	0.4036
**-7.7**		
control vs. 100µM LL	ns	0.993
control vs. 1mM LL	ns	0.7017
control vs. 10mM LL	ns	0.8196
**-7.4**		
control vs. 100µM LL	ns	0.8586
control vs. 1mM LL	ns	0.9763
control vs. 10mM LL	ns	0.4896
**-7.1**		
control vs. 100µM LL	ns	0.9627
control vs. 1mM LL	*	0.0103
control vs. 10mM LL	***	0.0001
**-6.8**		
control vs. 100µM LL	ns	0.5947
control vs. 1mM LL	ns	0.1132
control vs. 10mM LL	****	0.0001

Supplementary Figure 1. NE 52-QQ57 has no effect on cardiovascular variables and neurovascular coupling in anaesthetised rats.

A. Peripheral application of NE 52-QQ57 does not affect the arterial blood pressure and heart rate. Increasing doses of NE 52-QQ57 were injected i.p.; dose is shown as cumulative. two-way ANOVA revealed no significant differences between baseline and any other point.

B. Cortical CBF and BOLD responses to somatosensory stimulation are not affected by administration of NE 52-QQ57 (20mg/kg).

Top – pseudo-coloured images of BOLD signals evoked by sensory stimulation before and after systemic administration of NE 52-QQ57.

Bottom - dynamics of CBF and BOLD responses before and after administration of NE 52-QQ57.

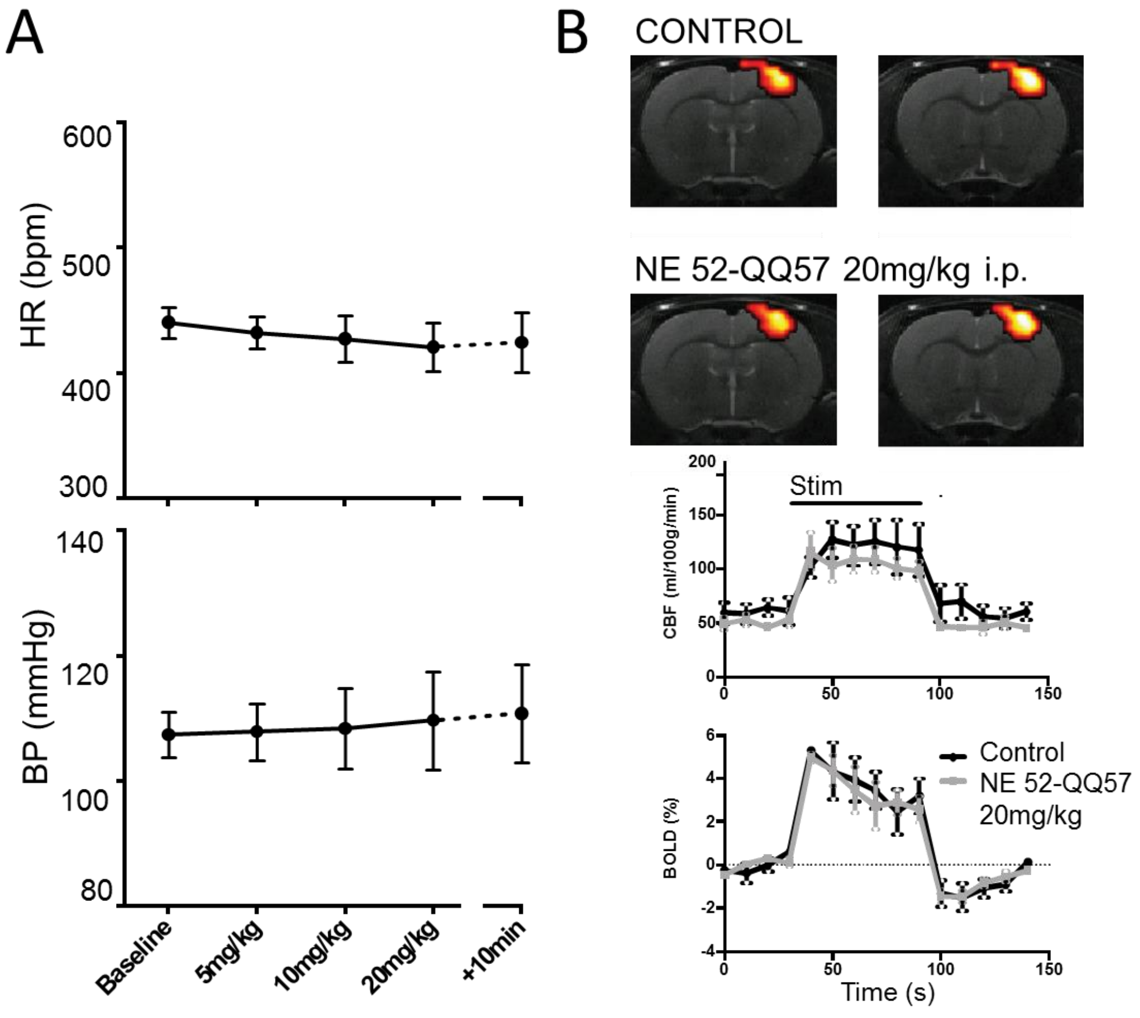

Supplementary Figure 2. GPR4 expression in a mouse brain as measured by fluorescent *in situ* hybridisation in A: blood vessel (magnified from the main Figure 1J), scale bar 10µm; B: retrotrapezoid nucleus (magnified from the main Figure 1Q), scale bar 50µm; C: locus coeruleus (magnified from the main Figure 1N), scale bar 50 µm. Green arrows indicate vessels.

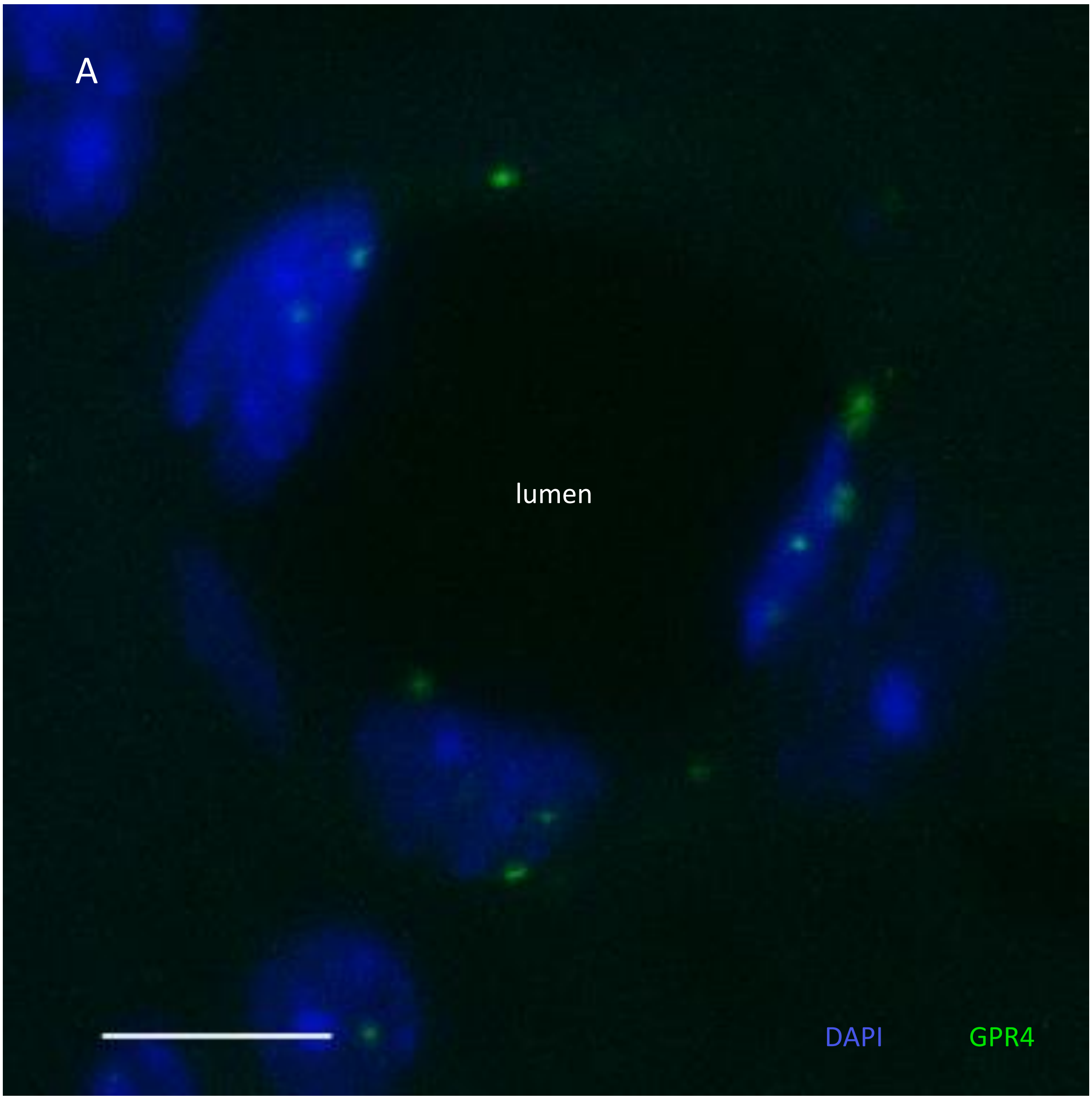

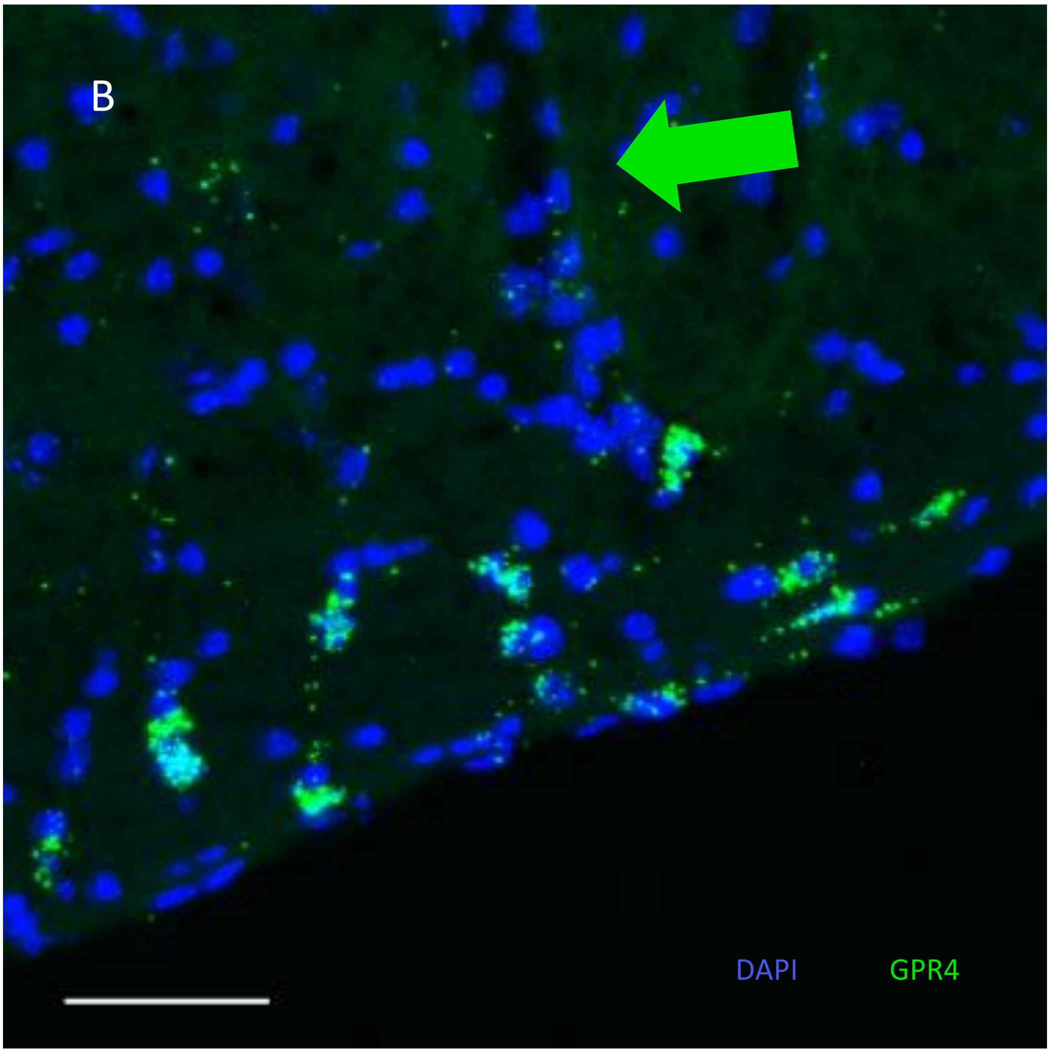
B
DAPI
GPR4

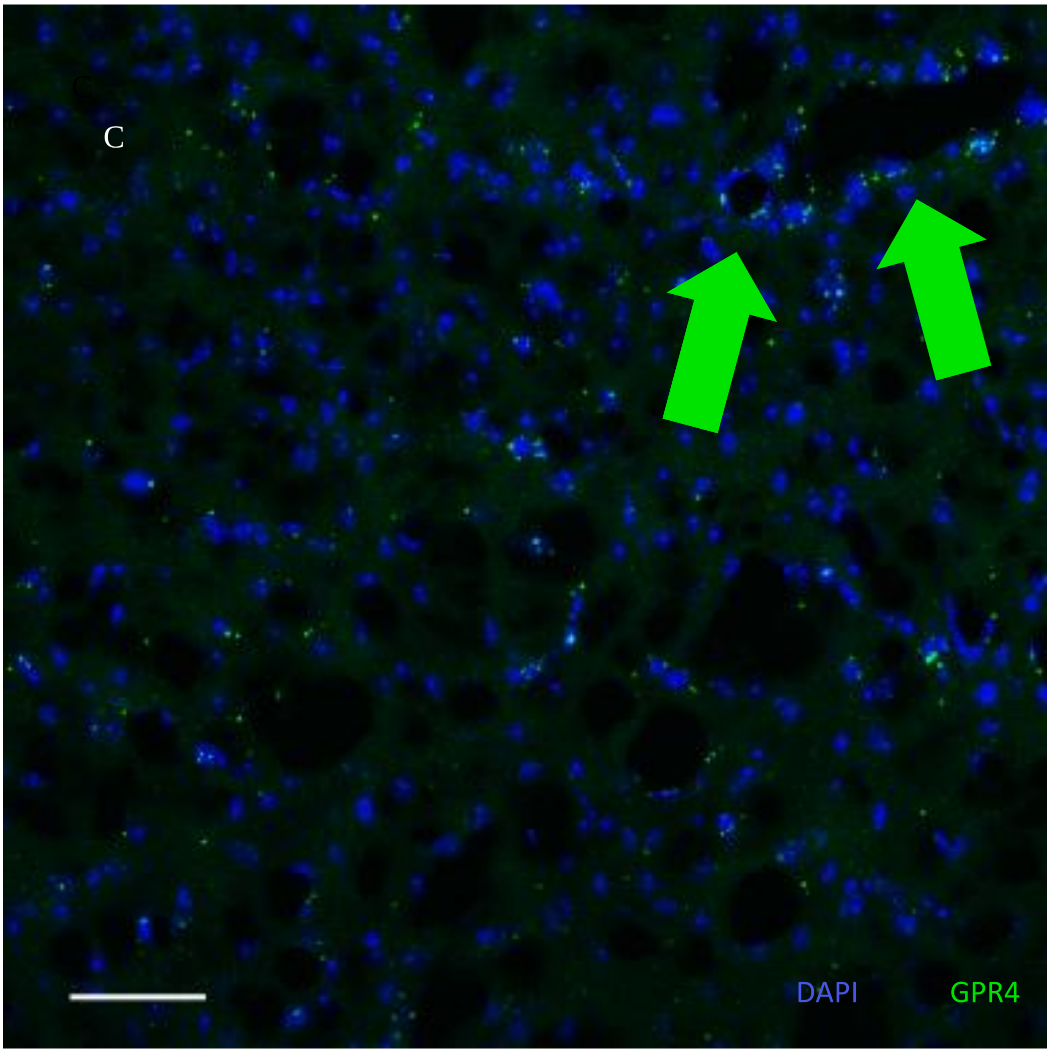
C
DAPI
GPR4
